# Understanding the Key to Targeting the IGF Axis in Cancer: A Biomarker Assessment

**DOI:** 10.3389/fonc.2015.00142

**Published:** 2015-07-08

**Authors:** Kunal Amratlal Lodhia, Piyawan Tienchaiananda, Paul Haluska

**Affiliations:** ^1^Department of Oncology, Mayo Clinic, Rochester, MN, USA

**Keywords:** insulin receptor, insulin-like growth factor receptor, IGF binding proteins, biomarker discovery, IGF system and signaling, insulin receptor substrate proteins, endocrine system diseases, targeted therapies

## Abstract

Type 1 insulin like growth factor receptor (IGF-1R) targeted therapies showed compelling pre-clinical evidence; however, to date, this has failed to translate into patient benefit in Phase 2/3 trials in unselected patients. This was further complicated by the toxicity, including hyperglycemia, which largely results from the overlap between IGF and insulin signaling systems and associated feedback mechanisms. This has halted the clinical development of inhibitors targeting IGF signaling, which has limited the availability of biopsy samples for correlative studies to understand biomarkers of response. Indeed, a major factor contributing to lack of clinical benefit of IGF targeting agents has been difficulty in identifying patients with tumors driven by IGF signaling due to the lack of predictive biomarkers. In this review, we will describe the IGF system, rationale for targeting IGF signaling, the potential liabilities of targeting strategies, and potential biomarkers that may improve success.

## Background

The type 1 insulin-like growth factor receptor (IGF-1R) and its signaling components are required for the development of the malignant phenotype, and low IGF bioactivity protects against the development of clinical cancers (1). IGF-1R overexpression has been consistently shown in multiple types of cancer, including pediatric and epithelial cancer and sarcomas (2, 3). The first assessment of IGF-1R targeted treatment used αIR-3, a mouse monoclonal antibody (mAb), which blocked IGF-1 binding to IGF-1R and inhibited growth of estrogen-independent breast cancers *in vitro* and *in vivo* (4–6). This has made IGF-1R a very attractive target, and currently two main therapeutic approaches are being developed: anti-IGF-1R monoclonal blocking antibodies and small molecule tyrosine kinase inhibitors (TKIs) (7–9). IGF-1R antibodies function by blocking interactions between the ligand and receptor, subsequently leading to receptor internalization and degradation (10). Additionally, IGF-1R mAbs result in insulin receptor (INSR) downregulation in cells expressing IGF-1R-IR hybrid receptors (HR) (10, 11). IGF-1R TKIs act by competing with ATP for binding in the kinase domain of IGF-1R and INSR, due to the highly conserved structure (7).

Eight IGF-1R targeting mAbs have been evaluated clinically, including AVE1642, BIIB022, cixutumumab, dalotuzumab, figitumumab, ganitumab, robatumumab, and R1507 (7). Clinical trials using these antibodies have shown limited activity in uncommon tumors such as ovarian carcinomas and Ewings sarcomas, as well as thymonal and adrenocortical carcinomas, but little benefit as single agent in common cancers. In addition, IGF-1R antibodies were recently reported to have single agent activity in recurrent ovarian cancer (12). However, in combination with other forms of therapy such as chemotherapy or other targets agents, they have shown some evidence of clinical benefit (7, 9, 13). For example, the combination of IGF-1R antibody with chemotherapy has led to significant increase in response rates, with little added toxicity in non-small cell lung cancer (NSCLC) (14, 15). However, this approach failed to prolong survival in unselected patients, leading to premature discontinuation of Phase 3 trail (16).

Six small molecule inhibitors have been evaluated clinically: BMS-754807, Insm-18 (NDGA), XL-228, OSI-906 (linsitnib), AXL1717 (PPP), and KW-2450 (7). Small molecule inhibitors may offer several potential advantages over blocking antibodies. Inhibitors can be administered orally and have a shorter half-life than antibodies, in the order of hours rather than days (17). This property can be exploited to allow for dosing flexibility, which can be helpful in optimizing scheduling IGF-1R inhibition with other agents. Moreover, the small molecule inhibitors target the tyrosine kinase domain of the IGF-1R, which shares a high degree of homology with the insulin receptor kinase domain. This allows for not only targeting of IGF-1R but also the INSR isoform, insulin receptor A (INSR-A), which can mediate tumor growth. Initial clinical experience suggests that co-inhibition of the metabolic isoform of the INSR, insulin receptor B (INSR-B), is tolerable (18).

It is possible that a reason for lack of success in targeting the IGF pathway is the lack of absolute dependence on IGF signaling for tumor survival. Alternatively, it is more likely that we simply have not selected the correct pathways for clinical investigation. This would be supported by anecdotal evidence of antitumor activity. As such, the key issue for successful clinical use of IGF-1R inhibiting drugs is the need to identify biomarkers that predict sensitivity to IGF-1R inhibition, in order to better select patients that would benefit from single agent or combine IGF-1R inhibition effectively with chemotherapy, radiotherapy, or other targeted agents.

## Insulin Receptor and Insulin-Like Growth Factor System

**Figure 1:**
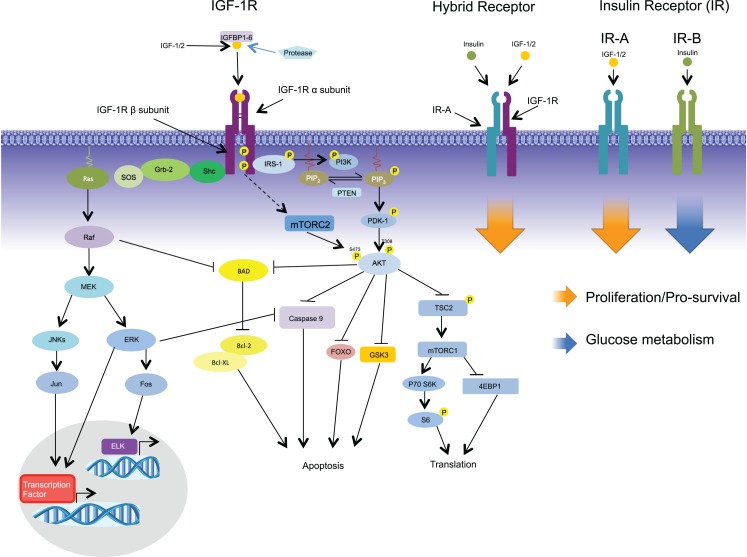
**Signaling via the IGF-1R begins with binding of its ligands, IGF-1, IGF-2, or insulin, to the extracellular α-subunit, and this in turn leads to phosphorylation of the intracellular β-subunits**. Recruitment of the adaptor protein IRS-1 to the activated receptor initiates signaling via the PI3K-AKT pathway leading to such cellular responses as protection from apoptosis, proliferation, and translation. Recruitment of adaptor protein SHC leads to activation of the RAS-MEK pathways leading to changes in gene expression. In addition to the formation of homodimers, IGF-1R/IR-A hybrid receptors can be formed comprising of two half receptors; signaling via the hybrid receptors leads to proliferation. In contrast to IR-A, IR-B binds primarily to insulin initiating glucose metabolism.

The IGF system includes three ligands (IGF-1, IGF-2, and insulin) and two homolog receptors, which are IGF-1R and INSR. Each of these receptors are heterodimeric proteins consisting of two extracellular α subunits and two transmembrane β subunits (19). There are two splice variants of the INSR: INSR-A, which is missing a 12 amino-acid sequence from exon 11, and the full length isoform, INSR-B (20). INSR-B is predominately expressed in insulin target tissue: liver, adipose tissue, and muscle (21, 22). INSR-A is expressed in embryo and fetal tissue; therefore, it is called “the fetal INSR isoform” (23–25). Both INSR isoforms have the same affinities with insulin; however, INSR-B primarily mediates metabolic effects (26), whereas INSR-A promotes cell growth, proliferation, and survival (27, 28). IGF-2 binding affinity to INSR-A is very high when compared with INSR-B. In addition, the affinity of IGF-1 to INSR-A is 10-fold higher than INSR-B (24, 29). Proinsulin, which has inactive metabolic function, was recently found to be able to stimulate INSR-A with the same affinity as insulin and induced similar biological effect (30). The role of proinsulin in malignancy is still under investigation. The major downstream cascades of IGF system are PI3K/AKT/mTOR and Ras/Raf/MEK/ERK (Figure 1) (31–36). Activation of INSRs also contributes to the downregulation of PTEN, enhancing PI3K activation (37). The action of insulin on signal transductions through INSR-A is somewhat different from those elicited by IGF-2 in terms of regulating certain gene and intracellular mediators (38, 39). The mitogenic effect from IGF-2 binding to INSR-A (IGF-2/INSR-A loop) is more pronounced than insulin (39). Moreover, INSRs have signaling crosstalk with β-catenin/Wnt pathway involved in proliferation and differentiation program (40).

INSRs are overexpressed in several malignancies including breast, colon, lung, ovary, and thyroid cancer (24, 41, 42). Additionally, INSR-A is the predominant isoform expressed in a variety of tumors such as breast, colon, ovarian, endometrium, and lung cancer (24, 27, 43–45). In ovarian cancer cell lines, INSR-A also preferentially expressed and mediated signaling in response to low dose insulin and IGF-2 (45). Moreover, activation of IGF-2/INSR-A loop promotes invasion and metastasis in choriocarcinoma (46), while downregulating of INSRs reduces cell proliferation, angiogenesis, and metastasis in cancer cells both *in vitro* and *in vivo* (27, 47). In colorectal cancer, INSRs expression was found only in blood vessels at the peritumoral region, but not in normal tissue, suggesting INSRs may be involved in promoting angiogenesis (48). These data suggest that INSRs itself can promote cell transformation and tumor development, and that INSR-A is an important component of IGF signaling.

Insulin growth factor signaling may also have a crucial role in cancer stem cell survival. Work by Bendall and colleagues identified IGF-2 as having a direct role in the survival and self-renewal ability of pluripotent human embryonic stem cells (49). IGF-2 has also been shown to promote stemness though INSR-A in neural stem cells in presence of IGF-1R blockade (50, 51). Similar observations were made in thyroid cancer and hepatocellular carcinoma (HCC), where undifferentiated cancer cells preferentially expressed higher levels of INSR-A and produced higher levels of IGF-2 in order to activate the autocrine loop (41, 52). In addition to the presence of INSR-A, a decreased ratio of INSR-A:INSR-B was reported to be associated with thyroid cancer, colon cancer, and HCC cell line differentiation to a more typical epithelial phenotype (53–55). Collectively, IGF-2/INSR-A autocrine loop has been considered as an important signaling pathway in cancer stem cell biology, and INSR-A:INSR-B ratio may be important in the pluripotent phenotype of cancer cells.

Recent nucleotide sequencing and gene expression analysis reported an increased INSR-A:INSR-B ratio in cancer tissue when compared with normal tissue in breast, colon, lung, endometrioid uterine, HCC, clear cell renal, and papillary cell renal carcinomas (56). Furthermore, the prevalence of high INSR-A:INSR-B ratio was found in >93% brain tumors and acute myeloid leukemias (56). The finding of increased INSR-A:INSR-B ratio was confirmed by quantitative PCR in breast, NSCLC, prostatic, HCC, and seminoma (56–59). In these reports, INSR-A mRNA expression level was similar in both tumor and adjacent healthy tissue, while INSR-B mRNA expression level was significantly decreased in tumor when compared to healthy tissue (44, 56, 57, 59, 60). Activation of the EGRF/ERK pathway was associated with increased INSR-A:INSR-B ratio in HCC (61). In addition to expression of INSRs, increased INSR-A:INSR-B ratio may contribute to the proliferation of various cancer types by both activation of IGF and EGFR pathway, raising the possibility of its value as a biomarker.

## Hybrid Receptors

IGF-1R and INSRs share approximately 85% homology, with maximal homology in the kinase domain (62). Moreover, these two receptors can form HR A and B (HR-A and HR-B), depending on the respective associated INSR isoform (63, 64). This adds an additional level of complexity in understanding the signaling state of the IGF pathway. These HRs randomly assemble with the two INSR isoforms and IGF-1R with the same efficiency (29, 65–68). Thus, a marked increased in INSR leads to the formation of HRs rather than IGF-1R homodimers. Both HR-A and HR-B have high affinities to IGF-1 and to a lesser extent for IGF-2 (65, 68). Cancer cells often express both IGF-1R and INSR-A that contribute to the activation of IGFs signaling by IGF-1 and to a lesser extent by IGF-2 (42, 66, 69). The majority of breast cancer specimens expressed HRs rather than IGF-1R (66). *In vitro* experiments treating breast cancer cell lines overexpressing HRs with IGF-1 induced greater auto-phosphorylation compared to IGF-1R homodimers (66). Overexpression of INRSs and HRs was also shown on malignant prostate epithelial when compared to benign prostate epithelium and this was shown to correlate with a higher Gleason score associated with poorer prognosis (58).

## INSRs Signaling as a Resistant Mechanism to IGF-1R Inhibitory Agents

Insulin receptor expression may lead to a compensatory mechanism for tumor treated with IGF-1R targets agents. *In vitro* work by Zhang and colleagues demonstrated that downreguation of the IGF-1R in breast cancer cell line leads to the increased insulin sensitivity (70). When IGF-1R was blocked, INSR-mediated ERK/AKT activity was increased in response to insulin at concentrations near to physiologic levels and enhanced INSR-A homodimer formation (71–73). In Ewing’s sarcoma cell lines that acquired resistance to anti-IGF-1R mAbs and TKIs, overexpression of INSR-A homodimer and/or IGF-2 production was observed (74). Vincent and colleagues used a NSCLC model and colleagues demonstrated that dual inhibition of IGF-1R and INSRs more effectively reduced NSCLC cell proliferation in cells with high and low IGF-1R:INSR expression ratio (75). In this report, INSRs mediated NSCLC proliferation when only IGF-1R was blocked, suggesting that the resistant mechanisms to IGF-1R inhibitory agents may be the ability of tumor cell to switch from IGF-2/IGF-1R to IGF-2/INSR-A dependency (75). Moreover, IGF-1 might have a negative feedback at pituitary level causing growth hormone induced hyperinsulinemia consequently to INSR-A activation (76, 77). Altogether, INSRs play an important role in the mitogenic effect of IGFs signaling pathway and may contribute mechanisms of resistance to IGF inhibitory agents.

## INSR-A:INSR-B Ratio as a Cancer Biomarker

In breast cancer, increased INSR-A:INSR-B ratio, measured by mRNA expression, has been reported in estrogen receptor positive (ER+) and negative (ER-) primary untreated breast tumors, and ER+ hormone refractory breast tumors (78). In addition, high INSR-A:INSR-B ratio was significantly higher in luminal B than luminal A subtype. Increased INSR-A:INSR-B in breast cancer also correlated with high proliferation index by OncotypeDx and FAK activation (44, 60). Ratio of INSR-A:INSR-B may serve as a useful biomarker to predict prognosis in breast cancer. In contrast to breast cancer, in squamous cell lung carcinoma, high INSR-A:INSR-B ratio was associated with lower epithelial-mesenchymal transition (EMT) gene signature and longer survival (56). A recent case control study of colorectal adenoma in patients found no difference in the total INSR mRNA; however, in patients with high plasma insulin, increased INSR-A:INSR-B ratio was associated with increased likelihood of having adenomas (79). Therefore, the usefulness of INSR-A:INSR-B ratio likely varies by tumor type and should be evaluated separately.

## INSRs and HRs as Cancer Biomarkers

A high mRNA level of INSRs and phosphorylated INSRs is associated with poor prognostic features such as high-grade, advanced stage, and deep invasion in endometrial cancers (80). Furthermore, immunohistochemical staining of NSCLC, especially squamous cell carcinoma, demonstrated that overexpression of INSRs by immunohistochemistry (IHC) correlated with poor overall survival (OS) (81). In early stage or lymph node negative breast cancer, high expression of INSRs was associated with favorable progression free survival (PFS) and OS (82, 83). Contrary to a result from unspecified breast cancer patients, total INSRs and phosphorylated IGF-1R/INSRs overexpression by IHC was related to poor OS in all breast cancer subtypes including those with acquired resistance to tamoxifen (84). These inconclusive results of INSRs as potential prognosis biomarkers in breast cancer warrant the need for further studies.

Anti-IGF-1R mAbs have demonstrated potent growth inhibition in breast cancer cell lines with a low HR:IGF-1R ratio (66). Subsequent studies in breast cancer examining high HR:IGF-1R demonstrated greater anti-tumoral activity using h7C10, a mAb targeting HR and IGF-1R, compared to mAbs targeting IGF-1R alone (85). High ratio of INSR:IGF-1R also conveyed resistance to the IGF-1R mAb cixutumumab in breast cancer cell lines (86). Hence, for breast cancer, INSR/HR:IGF-1R ratio may predict response to mAb against IGF-1R. *In vivo* data from INSRs knockout pancreatic neuroendocrine tumor cell lines demonstrated that lack of INSR increased sensitivity to cixutumumab (86). However, this was not seen in gastric cancer and HCC cell lines. A study by Kim et al. demonstrated that high level of IGF-1R and INSRs expression predicted favorable sensitivity to IGF-1R inhibition by the mAb figitumumab (87). Furthermore, transfection of low INSR expressing cells with an INSR-expression construct resulted in increased formation of HRs and enhanced growth inhibition from figitumumab (87). Finally, in this report, high HR expression was a strong predictive biomarker for figitumumab efficacy (87). The role of INSR/HRs expression as potential predictive biomarker to mAb to IGF-1R is needed to be further evaluated because of conflicting results.

Small molecule TKIs offer an approach to target both IGF-1R and HR simultaneously. The dual IGF-1R/INSR small molecule inhibitor OSI-906 demonstrated anti-proliferative effects in HCC cells lines overexpressing INSR and IGF-2 (88). Moreover, high mRNA expression of IGF-2/INSR correlated with EMT gene signature, which also was associated with increased sensitivity to OSI-906 (88). Taken together, overexpression of IGF-2/INSR predicts the EMT phenotype and response to OSI-906 in HCC cell lines, and offers a method targeting IGF-1R, INSR, and HR due to the high degree of homology in the kinase domain of these receptors.

## Insulin like Growth Factor Binding Proteins

There are six high affinity superfamilies of IGF binding proteins (IGFBPs) that function as key regulators of the IGF pathway (Figure 1) (89). IGFBPs are an important consideration in IGF signaling potential, as they are key regulators in the bioavailability of circulating IGF ligands (90). More recently, IGFBPs have intracellular roles in regulating growth and survival, and intranuclear roles in transcription regulation, induction of apoptosis, and DNA damage repair (91–93). In circulation, IGFBPs 1–5 has the same affinities to IGF-1 and IGF-2, while IGFBP-6 has a binding preference for IGF-2 (90). The binding affinity of IGFBPs to IGF ligand is similar to the binding affinity of IGFs to IGF-1R (94–97). The key function of circulation IGFBPs, especially with IGFBP-3 and to a smaller extent IGFBP-5, is to form ternary complexes, including IGFs, IGFBPs, and the acid-labile subunit (ALS) (98). These complexes not only account for the major circulating IGFs but also increase the half-life of unbound IGFs from a few minutes up to 16–24 h (98–100). Since, IGFBPs have a sequestration affect that limits bioavailability of circulating IGFs and competitively inhibits IGFs to bind with IGF-1R at pericellular region, leading to the tumor suppressor action of IGFBPs.

IGFBP-3 and IGFBP-5 are also well established as inhibitory effect on tumor growth via an IGF-independent mechanism (101–103). The type V transforming growth factor-β (TGF-β) receptor (also known as low density lipoprotein receptor-related protein 1; LRP1) is implicated as a binding receptor of IGFBP-3 and possibly, to a lesser extent, IGFBP-5 (104). TGF-β mediates growth inhibition by dephosphorylating insulin receptor substrate 2 (IRS-2) and stimulates Smad 2/3 (104–106). Moreover, at cell surface, the putative IGFBP-3 receptor (also known as transmembrane protein 219; TMEM219) is hypothesized to be death receptor, stimulating caspase 8 dissociating from its cytoplasmic tail upon ligand binding, and promoting apoptosis (107, 108). In endoplasmic reticulum, IGFBP-3 can form a complex with the chaperone protein GRP78 that induces apoptosis by competing with caspase 7 for GRP78 binding (109). The IGFBP-3-GRP78 complex has also been demonstrated to augment autophagy as a result of cellular stress; however, the mechanism for these is still unknown (109, 110). In addition, IGFBPs can be internalized to nucleus and have interactions with nuclear hormone receptors, including retinoid X receptor, retinoic acid receptor, and the vitamin D receptor (93, 111). Activation of these receptors induces transcription of IGFBP-3, IGFBP-4, IGFBP-5, and IGFBP-6, which results in growth inhibition (110–116). For example, IGFBP-3 can bind to retinoid X receptor, which induces pro-apoptotic transcription activity (93). IGFBP-3 is required for the formation of EGRF and DNA dependent kinase (DNA-PK) complex in nucleus to initiating DNA repair after DNA double-stand breaks following chemotherapy (92). IGFBP-3 is also directly phosphorylated by DNA-PK, which stimulates its binding to EGFR and leads to induction of apoptosis (117, 118). IGFBP-6 enhances SEMA3B, a member of class 3 semaphorins activity to inhibit vascular epithelium growth factor (VEGF). Therefore, expression of IGFBP-6 can result in suppression of angiogenesis (119).

Furthermore, mounting evidences are shown that IGFBPs may not be purely regulatory, but may also have oncogenic potential. IGFBP-1, IGFBP-2, and IGFBP-6 stimulate cell migration and metastasis by interacting with α5 integrin and prohibin 2 (120–122). Similarly, in activated Kras background, overexpression of IGFBP-2 promotes tumor growth (123). IGFBP-2 also positively regulates β-catenin in Wnt signaling and integrin β1-ERK, leading to pro tumorigenic effects (124, 125). In addition to intracellular activity, the nuclear import of IGFBP-2 increases activation of VEGF transcription resulting in increased angiogenesis (126). In the context of breast cancer, IGFBP-3 and IGFBP-5, secreted by carcinoma-associated fibroblasts (CAFs), inhibit detachment-induced cell death, known as anoikis, via regulation of ERK-MAPK activation (127).

The dichotomous effect of IGFBP-3 and IGFBP-5 may be explained by the “sphigolipid rheostat,” which determines cell fate and their interaction with microenvironment. Ceremide and sphigosine mediate cell cycle arrest and cell death by autophagy; in contrast, sphigosine-1-phophate (S1P) promotes cell survival and proliferation (128). Sphigosine kinase (SK) generates S1P from sphigosine. S1P can transactivate various growth factor receptors, including EGFR and IGF-1R, by transportation to extracellular space then activating S1P receptor (129, 130). Exogenous IGFBP-5 stimulates SK *in vitro*, then increases the level of S1P that may contribute to survival effect; however, when fibronectin is present in cell culture, the survival effect of IGFBP-5 is lost (131). On the other hand, IGFBP-3 promotes ceramide induced cell death when cells are grown *in vitro*; however, when cells are grown on fibronectin, IGFBP-3 actions were reversed (131). IGFBP-3 also activates SK and increases phosphorylation of EGF in a triple negative breast cancer cell line (132).

## IGFBPs as Potential Predictive and Prognostic Biomarkers

High level of circulating IGFBP-1 was associated with poor all-cause mortality and cancer specific death in colorectal cancer patients (CRC) (133, 134). However, in a large Phase II study, this putative prognostic biomarker failed to be confirmed (135). In this and subsequent other studies, serum IGFBP-1 also did not demonstrate a predictive value for cancer risk in CRC, NSCLC, and endometrial cancer (136–140). In prostate cancer, high level of IGFBP-1 was associated with increased cancer risk and a shorter time to castration resistant prostate cancer from androgen deprivation therapy (ADT) and reduced OS (141, 142). This suggests that in prostate cancer, IGFBP-1 can both serve as a prognostic and predictive biomarker. In HCC, on the other hand, low tissue expression of IGFBP-1, assessed by IHC, was associated with decreased OS (143). With regards to treatment with the mAb cixutumumab, high serum IGFBP-1 predicted improvement in PFS and OS in HCC patients (144). However, high circulating levels of IGFBP-1 failed to predict response to mAb against IGF-1R, ganitumab, in CRC (135). There are conflicting data concerning the predictive and prognostic value of IGFBP-1 as a biomarker, and further studies in a variety of tumor types are necessary.

There is strong evidence of an association between gliomas and IGFBP-2. Both high levels of serum IGFBP-2 and high tissue expression IGFBP-2 by both IHC and mRNA levels correlate with poor PFS and OS in gliomas, including glioblastoma multiforme (GBM) (145–149). Similarly, associations exist in CRC, where high serum IGFBP-2 correlated with increased mortality rate (135, 150, 151). Nonetheless, the prognostic potential of circulating IGFBP-2 in CRC was not demonstrated in other studies (134, 137). In addition, circulating IGFBP-2 did not predict response to ganitumab in CRC patients (151). High serum levels and high IHC score of IGFBP-2 in NSCLC were also associated with poor OS (152, 153). In contrast, high serum level of IGFBP-2 was associated with better OS in adrenocortical carcinoma (154). Low serum level of IGFBP-2 in advanced pancreatic cancer predicted the improvement of OS from ganitumab and gemcitabine (155). The role of IGFBP-2 as a potential biomarker for prostate cancer was evaluated in a large case control study; however, serum IGFBP-2 did not predict risk of prostate cancer (156). There was no association between serum level of IGFBP-2 and cancer risk in endometrial and ovarian cancer (138, 157). IGFBP-2 was shown to contribute to ovarian cancer cells invasion *in vitro* (158). Clinically, an elevated serum level of IGFBP-2 was shown to correspond with poorer prognosis, suggesting prognostic value of IGFBP-2 in ovarian cancer (159). Furthermore, *in vitro* treatment of ovarian cancer cells with IGFBP-2 was shown to stimulate cell growth and potentiated the activation of multiple signaling pathways involved in cell proliferation (160). Similar to IGFBP-1, studies examining the prognostic and predictive value of IGFBP-2 as a biomarker are conflicted and require further studies to clarify its value in specific tumor types.

The most abundant IGF binding protein is IGFBP-3, and thus, many studies have focus on IGFBP-3 level and/or its ratio to the IGF-1 ligand, which may represent the inactive fraction of circulating IGF ligand. In one large case control study, high serum IGFBP-3 was associated with lower risk of CRC; however, several other studies failed to demonstrate this same association (133–137, 140, 161). High serum IGFBP-3 in CRC patients also did not predict response to ganitumab, however did correlate with poor PFS and OS (151). In breast cancer, the data regarding IGFBP-3 are conflicting. Some studies have reported that high serum levels of IGFBP-3 are associated with increased risk of breast cancer in pre- and or post-menopausal women (162, 163); however, several studies were unable to confirm this finding (164–167). The ratio between IGF-1 and serum IGFBP-3 was associated with increased mortality, suggesting a potential prognostic biomarker value of this ratio in breast cancer (167). In addition, levels of IGFBP-3 in breast cancer tissue, determined by IHC but not mRNA levels, were associated with poor OS (168, 169). A small cohort study in breast cancer examining serum IGFBP-3 levels before and 1 week after palliative chemotherapy demonstrated that patients who showed decreased IGFBP-3 levels after treatment showed poorer OS (170). In prostate cancer, high serum level of IGFBP-3 correlated with increased cancer risk and cancer specific death (156, 171–173). Expression of nuclear IGFBP-3 in prostatic cancer tissue was also associated with decreased PFS time (174). However, other studies have failed to confirm the predictive value of circulating IGFBP-3 for increased risk of prostate cancer (175, 176). In NSCLC, data more consistently demonstrate that high levels of serum IGFBP-3 are associated with a better prognosis for PFS and OS (177–179). Moreover, decreased expression of IGFBP-3 from stage I NSCLC tissue was associated with poor PFS (180). In other cancer types, specifically ovarian, endometrial, pancreatic, gastric, and renal cancer, no association was demonstrated between serum IGFBP-3 and cancer risk (157, 181–187). In other cancer types, specifically in squamous cell carcinoma of esophagus and tongue, gastric cancer, HCC, and ovarian endometrioid cancer including, low tissue expression of IGBP-3 was associated with poor disease outcome (PFS or OS) (188–192). In GBM tissue, high IHC expression of IGFBP-3 was related to poor OS (193). High serum level of IGFBP-3 in advanced pancreatic cancer predicted the improvement in OS as a result of combined treatment with ganitumab and gemcitabine (155). In contrast, circulating IGFBP-3 did not correlate with a response to ganitumab in CRC, and the combination of cixutumumab and the mTOR inhibitor, temsirolimus, in other solid tumor patients (151, 194).

In breast cancer, high IHC staining for IGFBP-4 was associated with longer PFS and OS (195). However, serum IGFBP-4 was not associated with cancer risk for NSCLC (139). Additionally, a small study demonstrated that IGFBP-4 levels were inversely correlated with survival across all stages of epithelial ovarian cancer (196). There has been no work publishing relationship between IGFBP4 and response to IGF targeting agents. Unlike the other IGFBPs, few studies exist, examining IGFBP4 as a biomarker and might represent an under-explored biomarker for predicting response to IGF-1R targeted therapies.

Low circulation IGFBP-5 was associated with poor PFS in NSCLC (139). In breast cancer, high tissue expression by IHC or mRNA levels correlated with decreased PFS and OS (195, 197, 198). Furthermore, a high mRNA ratio of IGFBP-5/IGFBP-4 from patients’ tissue enhanced the power of its poor prognostic value, and also predicted resistance to the IGF-1R and INSR TKI, BMS-536924, in a breast cancer cell line (197). After tamoxifen treatment, low IGFBP-5 IHC expression correlated with poor OS (199). In breast cancer patients, high serum levels of IGFBP-5 correlated with improved time to treatment failure of cixutumumab (200). Collectively, the tissue level of IGFBP-5 in breast cancer may be both prognostic and predictive biomarker for tamoxifen and IGF inhibitory agents. IGFBP-5 overexpression by IHC in urothelial carcinomas of upper urinary tracts and urinary bladder was associated with poor prognosis (201). Recently, low IGFBP-5 mRNA expression in 93 cancer cell lines predicted a good response to figitumumab *in vitro* (202). Hence, the prognostic and predictive value of IGFBP-5 is encouraging and further study evaluating IGFBP-5 mRNA levels as a potential biomarker for IGF-1R mAbs is warranted.

There is no study that directly investigated IGFBP-6 as a potential biomarker. As a biomarker, there is no consistent evidence from pre-clinical and clinical studies suggesting the value of IGFBPs to predict outcome or response to IGF targeted treatments. It may due to the considerable complexity of the IGF-IGFBPs system and their interaction with other pathways. Despite contradictory results of IGFBPs, further studies are needed, investigating the potential prognostic biomarker value that might need to be tailor for each IGFBP and cancer type.

## Nuclear IGF-1R

It is now well recognized that RTKs can translocate to the nucleus, and this was first shown for EGFR (203–205). Nuclear EGFR is capable of directly regulating gene transcription and also interacts with DNA-dependent protein kinase catalytic subunit (DNA-PKcs), a protein central to DNA repair by non-homologous end joining (NHEJ) (204, 206). Once inside the nucleus, EGFR functions as a co-transcription factor for several genes involved in cell proliferation and angiogenesis, and as a tyrosine kinase to activate and stabilize proliferating cell nuclear antigens and DNA dependent protein kinases (207). More recently, IGF-1R has been shown to translocate to the nucleus in cancers for the prostate, renal, breast, soft tissue sarcomas, and Ewing sarcomas (208–210).

Following activation, IGF-1R is known to be internalized by clathrin- and caveolin-mediated endocytosis (211, 212). Upon internalization, IGF-1R is transported to recycling endosomes where it is either recycled and shuttled back to the cell surface or transported to lysosomes for degradation (213). Work by Aleksic and colleagues showed that IGF-1 can stimulate ligand-dependent internalization and nuclear translocation of full-length IGF-1R subunits in prostate cancer cells (208). Nuclear translocation was also demonstrated in melanoma cells by Sehat et al., who also reported that IGF-1R nuclear translocation requires SUMOylation on three evolutionarily conserved lysine residues (K1025, K1100, K1120) in the beta subunit (214). Typically, proteins that are transported to the nucleus contain a nuclear localization sequence (NLS), which allows binding to importins for translocation through nuclear pores (215). The IGF-1R does not contain an NLS; however, Packham et al. recently demonstrated that IGF-1R first associated with the dynactin subunit p150^Glued^, which transports the receptor to the nuclear pore complex, where it co-localizes with importin-β. Similarly, the function of nuclear IGF-1R is unclear. Sehat et al. conducted a ChIP-sequencing experiment and identified IGF-1R binding sites in the genome, but these were few (~500) and most were remote from known genes (214). Recent studies have shown that nuclear IGF-1R can bind to LEF1 transcription factor and increase the promoter activity of LEF1 target genes including cyclin D1, which might be an additional mechanism by which IGF-1R promotes proliferation (210). In addition, IGF-1R/INSR HR are reported to localize to the nucleus of corneal epithelial cells (216).

Work by Aleksic and colleagues showed that higher levels of nuclear IGF-1R were associated with poor prognosis in renal cancer, suggesting that nuclear IGF-1R contributes to an aggressive phenotype (208). Recent studies in HCC treated with the EGFR inhibitor gefitinib showed increased levels of nuclear IGF-1R following treatment (217). The IGF-1R nuclear translocation was enhanced under gefitinib treatment and increased in a dose-dependent manner. This suggests that nuclear IGF-1R translocation following gefitinib treatment may contribute to resistance to IGF-targeting agents (217). Studies in a small group of patients with sarcomas treated with several IGF-1R monoclonal antibodies found that exclusive nuclear IGF-1R was associated with better PFS and PS (218). This suggests that exclusive nuclear IGF-1R staining might serve as a predictive biomarker for sarcoma patients likely to benefit from IGF-1R mAb therapy (218). Initial studies of nuclear IGF-1R point toward a role in transcription and regulation of the cell cycle; however, the complex role of nuclear IGF-1R is yet to be fully understood and research identifying its function and regulation in the nucleus will provide for the development of rational combination treatment in cancers that develop drug resistance.

## Biomarkers Based on Genetic Alterations Outside IGF-1R Signaling

IGF-1R accomplishes a wide range of functions via a complex signaling cascade, which raises the possibility of identifying biomarkers outside the immediate IGF axis. To support this idea, a study was reported using an unbiased siRNA screen to identify factors that regulate sensitivity to IGF-1R inhibition in childhood sarcomas. This work used the small molecule IGF-1R inhibitor BMS-536924 and screened a library of 88 RTKs and 31 Insulin/IGF signaling pathways proteins and identified ribosomal S6 and macrophage-stimulating 1 receptor tyrosine kinase (MSTR1R) (219). In this report, BMS-536924 failed to block RPS6 activation in resistant sarcoma cell lines; however, this siRNA targeting RPS6 restored BMS-53924 efficacy (219). Recently, work by Gao et al. performed an unbiased siRNA screen to identify factors that regulate sensitivity to a IGF-1R inhibitor (AZ12253801) in prostate cancer cells and breast cancer cells (220). Using this approach, two candidate biomarkers were validated, Dsh Homolog DVL3, apart of the WNT signaling pathway, and RAD51 required to strand invasion step of homologous recombination (220, 221). In both these reports, inhibition or genetic manipulation of DVL3 or RAD51 increased the sensitivity of tumor cells to IGF-1R inhibition, representing a subset of patients who might benefit from IGF-1R inhibition. Taken together, these studies in childhood sarcomas, and prostate, and breast cancers suggest that more studies are needed to investigate the potential of components outside the IGF axis to provide valuable predictive biomarkers for IGF-1R inhibition.

In addition to these screens, the breast cancer susceptibility genes, BRCA1 and BRCA2, have recently been shown to regular IGF-1R expression or influence the downstream signaling. *In vitro* work in MCF7 breast cancer cells demonstrated that BRCA1 knockdown induces the expression of IGF-1 mRNA in an estrogen receptor α-dependent manner, which was shown to correspond with increased IGF-1R activation and signaling (222). In a study examining women with *BRCA1* and *BRCA2* mutation reported significant association in variants in *IGF1R* and *IRS1* in *BRCA1* mutant carriers and also variants in *IGFBP2* for *BRCA2* carriers (223). Taken together, this suggests that tumors harboring mutations or deletions in BRCA 1/2 genes might be more sensitive to IGF-1R blockade. Further studies examining BRCA1 and BRCA2 status as a predictive biomarker for IGF-1R inhibition are warranted.

## Expression Profiles

Given the complexity of growth factor signaling, it is conceivable that there might not be a single predictive biomarker but rather a combination of multiple factors. The use of a gene expression patterns has been previously reported in breast cancer, in which IGF-1 treated MCF-7 cells were profiled for RNA transcription 3 and 24 h following treatment (224). In this report, IGF-1 treatment induced changes in the expression of genes associated with proliferation, metabolism, and DNA repair, and in particular, IGF-1 signature was enriched for transcriptional targets of PI3K/AKT/mTOR and Ras/MAP pathways (224). Similar profiles were observed in ER-negative breast tumors but also in 25% of ER-positive tumors, and tumors with this profile demonstrated poorer prognosis suggesting a predictive value in breast cancer (224). Subsequent work by Creighton et al. examined the transcriptional profile of the IGF-1R downstream signaling molecule PI3K and found that ER levels negatively correlated with PI3K activation levels both at the proteomic and transcriptional level (225). Treatment with a PI3K inhibitor resulted in decreased PI3K activity and increased ER expression *in vitro*, and thus might restore sensitivity to hormone therapy (225). This suggests that patients with ER-negative breast cancers might benefit from treatment with IGF inhibitory drugs, as IGF signaling is capable of directly influencing the activation of PI3K.

## Conclusion

Compelling pre-clinical data supported the use of IGF-1R targeted therapies; however, in unselected phase 2/3 clinical trails, IGF-1R targeted agents have shown little-to-no benefits. It is possible that the current generation of mAbs and small molecule inhibitors targeting the ligand binding domain or the intracellular kinase domain is not the correct approach, due to toxicity and hyperglycemia. Perhaps, a more effective approach is to limit the bioavailability of the ligands, IGF-1 and IGF-2, which inhibits IGF-induced IGF-1R and IR-A activation but does not affect insulin signaling. Such approach is currently being examined with the mAb MEDI-573 and BI 836845, which reduces proliferation and survival *in vitro* and *in vivo* (226, 227). The use of ligand mAbs as an approach to limit toxicity and hyperglycemia, while inhibiting IGF-1R/INSR-A mediated effects of tumor proliferation and survival, was supported by a Phase I trial using MEDI-573 (228). A total of 43 patients were treated with MEDI-573 at a dose greater than 5 mg/kg circulating levels of IGF-1 and IGF-II were fully suppressed (228). Moreover, BI 836845 enhanced the antitumor efficacy of rapamycin by blocking a rapamycin-induced increase in upstream signaling *in vitro* as well as in human tumor xenograft models in nude mice (226). Additionally, the lack of clinically effective IGF targeted therapies might be a result of the lack of predictive biomarker. Various pre-clinical studies have suggested that IGF targeted agents might benefit patients of specific molecular background. For example, patients with triple negative breast cancer or KRAS-mutant lung cancers might benefit from IGF-1R inhibitor drugs (229–231). The challenge in unlocking the potential for IGF targeted therapies lies in the need of predictive biomarkers.

## Conflict of Interest Statement

The authors declare that the research was conducted in the absence of any commercial or financial relationships that could be construed as a potential conflict of interest.
